# A new technique for tongue brushing and halitosis reduction: the X technique

**DOI:** 10.1590/1678-7757-2018-0331

**Published:** 2019-04-01

**Authors:** Ana Carolina de Souza Gonçalves, Marisol Corvino Nogueira Martins, Bruna Luísa de Paula, Paulo Henrique Weckwerth, Solange de Oliveira Braga Franzolin, Elcia Maria Varize Silveira

**Affiliations:** 1 Universidade do Sagrado Coração, Bauru, São Paulo, Brasil.

**Keywords:** Oral hygiene, Tongue, Halitosis, Microbiology, Treatment

## Abstract

The tongue is one of the primary sources of halitosis. The manual or mechanical removal of biofilm is known to decrease oral malodor.

**Objective:**

To evaluate a new tongue hygiene technique hereby referred to as “the X technique” and its effects on both halitosis and the number of microorganisms based on microbiological parameters and diagnostic features of the breath.

**Material and Methods:**

The study included thirty patients divided into a control group (patients without systematized guidelines of lingual hygiene, but who performed the mechanical cleaning of tongue dorsum, each in its own way), the 3R group (instructed to perform the movements of the X technique for 3 repetitions at each brushing), and the 6R group (instructed to perform 6 repetitions of the technique at each brushing). After two weeks, a new data collection was performed.

**Results:**

Patients in the 6R group presented the lowest score on the organoleptic assessment scale at the second consultation, followed by the 3R group and the controls. Regarding the self-perception of breath by the method of Visual Analog Scale (VAS), the control group did not perceive improvements in oral malodor; the results of the 3R group and the 6R group were similar.

**Conclusion:**

These results indicate that the X technique improves both measurements and perceptions of halitosis. Microbiological analyses revealed greatest reduction in the 6R group. The findings show that the X technique reduces both organoleptic scores and the number of bacterial colonies, and improves users’ perceptions of their breath.

## Introduction

The term “halitosis” comes from the Latin *halitos* (expired air) and *osis* (a pathological abnormality), it is a term that refers to unpleasant breath. Its non-oral etiology includes respiratory tract conditions, gastrointestinal and neurological disorders, various types of systemic diseases such as diabetes, certain types of carcinoma, hormonal changes such as menstruation and pregnancy.^1^ There is also physiological halitosis, known as morning halitosis, which appears after several hours of sleep and fasting, in response to the decomposition of food particles and bacterial agglomeration aggravated by reduction in salivary flow and pH.^2^

In most cases, however, the etiology of halitosis is intra-oral.^3^ Causes include certain types of foods, poor oral hygiene, periodontal disease, pericoronitis, ulcers, low salivary flow, food impaction, poorly fitting dental fillings, abscesses, prostheses, alcohol and nicotine consumption, infections in the oral cavity, and microbial metabolism in the dorsum of the tongue.^4^ ^-^ ^6^ Because they exhibit characteristics that facilitate proteolytic/putrefactive microbial activities, the tongue and the subgingival environment are considered the main sources of volatile sulfur compounds (VSCs), and high concentrations of these gases in the oral cavity may indicate breath abnormalities.^7^ It is important to note, however, that each of these sites produces different proportions of VSCs.^8^ The mechanical cleaning of these areas seems to significantly decrease the levels of VSCs and, consequently, to improve halitosis.^9^ It is likely that most adults will suffer from halitosis, at least occasionally, a prevalence which explains patients’ growing interest in seeking out professionals to diagnose and treat bad breath. Numerous microenvironments harbor halitosis promoting bacteria;^7^ ^,^ ^10^ however some researchers consider the back of the tongue to be the primary source of bad breath among both healthy patients and those with periodontal disease. While periodontitis is associated with halitosis, there is evidence that periodontally healthy people may also exhibit significant levels of oral malodor.^11^ The dorsum of the tongue is extensive and irregular, with cracks and papillary structures capable of retaining considerable amounts of substrates (dead leukocytes, shed epithelial cells), and it is an ideal site for the growth of microorganisms.^10^ ^,^ ^12^ ^-^ ^15^

These microorganisms are largely present on the posterior third of the dorsum of the tongue and are the main etiological factor in halitosis. Because the etiology of halitosis involves the presence of microorganisms, the therapy for reducing the coating on the tongue to improve halitosis consists of the mechanical reduction of said coating.^16^

The removal or eviction of the plaque on the tongue dorsum improves halitosis.^7^ ^,^ ^10^ Chemical-based reductions are also an option: mouthwashes such as those containing 0.12% chlorhexidine can reduce VSC levels by 43%, with a consequent decrease in organoleptic scores of up to 50%.^10^ ^,^ ^17^ ^,^ ^18^ Though the literature reports a variety of methods to promote tongue hygiene, there is still no consensus regarding the most effective technique for reducing halitosis. As a result of this lack of protocol, many patients adopt no lingual hygiene method at all. Their reasons also include a lack of information, difficulty in execution, and/or inability to purchase the devices available for this function.^9^ Studies have shown that tongue brushing is more effective in reducing halitosis than scraping, and patients generally prefer to clean the tongue using the toothbrush rather than scrapers.^19^ Nevertheless, there is still a need for scientific studies to standardize the techniques for the mechanical removal of the coating on the tongue through brushing, studies which will ideally show the advantages of the procedure and spread useful information.^9^ Based on these limitations, we sought to develop a new tongue brushing protocol that would provide a simple and accessible technique. The objectives of this study were to test a new tongue hygiene technique, hereby referred to as “the X technique”, and to evaluate its effects on oral malodor and on the number of microorganisms present on the tongue, using different diagnostic resources and microbiological parameters.

## Material and methods

Thirty patients (18 women and 12 men) were included in this study. They were 19 to 73 years of age (±43 years) and were referred from the teaching clinic in Bauru, São Paulo, Brazil. A screening was performed to exclude smokers, pregnant women, patients with cavities, patients with periodontal disease, and patients who had used systemic antibiotics in the three months prior to the study. The experimental groups were established as the control group (patients who did not receive information on tongue hygiene), the 3R group (patients who were instructed to brush the tongue using the X technique with three repetitions at each brushing), and the 6R group (patients who were instructed to brush the tongue using the X technique with six repetitions at each brushing), according to random selection to ensure homogeneity of the sample.

All patients received a soft toothbrush (Curaprox^®^ 5460 UltraSoft, Curaden, Switzerland) to specifically clean the dorsum of the tongue during the study period. After two weeks, a new data collection was performed.^20^ A written consent of all the participants involved in this study was obtained and subsequently submitted/approved by the local research ethics committee [Brazilian National Research Ethics Committee (CONEP) No. 1.045.212]. This study was conducted in full accordance with the World Medical Association Declaration of Helsinki.

### Implementation of the Organoleptic Assessment Method (OAM)

First, each patient was evaluated using the organoleptic assessment method performed by a trained and reliable examiner (Kappa 0.7). Each patient kept his or her mouth closed for 2 minutes. Next, with a distance of approximately 10 cm between the examiner's nose and the patient's mouth, each patient's breath was classified on a scale of 0 to 5, on which 0 represented the absence of odor, 1 represented the slight presence of odor, 2 represented a weak but clear odor, 3 represented moderate halitosis, 4 represented strong halitosis, and 5 represented extreme halitosis.^13^ ^,^ ^21^ ^-^ ^25^

### Measuring oral odor using the Visual Analog Scale (VAS)

Each patient's self-evaluation of oral odor was performed using a visual scale 10 cm in length. The left side of the scale read “no bad breath”, while the right side of the scale read “extreme bad breath”. The patient provided a score by marking a vertical line at the point where he or she considered his oral odor to lie based on self-perception.^26^

### Collection and analysis of tongue coating samples

The tongue coating samples from the dorsum of the tongue were collected using a No. 24 scalpel. Scraping was performed longitudinally beginning at the vallate papillae to the tip of the tongue for 10 seconds.^27^ Care was taken to avoid touching the teeth or the neighboring mucosa so that there would be no interference of adjacent biofilm. After the material was collected, the samples were immediately transported to the Microbiology Laboratory of *Universidade do Sagrado Coração* (USC) in a sterile isotonic sodium chloride (NaCl) solution (Linhamax^®^ 0.9 mg/ml Eurofarma Laboratórios SA, Ribeirão Preto, São Paulo, Brazil). The samples were sealed in test tubes with 9 ml of saline solution and 1 ml of the sample (tongue coating and saliva) in each tube. In the laboratory, the samples were diluted (1:1000) inside the flow chamber to avoid external contamination. For this process, 1 ml pipette tips (Goldlab 100/1000) and pipettes were used. The material was diluted, and 1 µl was poured into each sterile Petri dish (90×15 mm) containing brain heart infusion agar (Brain Heart Infusion, Kasvi^®^, São José do Pinhais, Paraná, Brazil – 37 g/l, distilled water, 121°C/15 min autoclave). After the inoculum was created, all of the Petri dishes were incubated in a Fanem^®^ conventional incubator at 37°C for 24 hours. After being incubated overnight, the Petri dishes were analyzed using a Phoenix^®^ CP 608 manual colony counter. Each colony identified was marked using a felt-tip marker. Colonies were considered independent when clearly separated from the others. The colonies in each Petri dish were counted by two different researchers for consistency and precision. All of the material used in the process, from the collection to the inoculation, was autoclaved prior to use (Phoenix Luferco^®^ vertical autoclave, Araraquara, São Paulo, Brazil). Two numerical results multiplied by the amount of dilution (10^3^) were obtained for each patient. This number represented the concentration of microorganisms *per* Petri dish (CFU/ml^-1^). It was associated with each patient's OAM and VAS score as part of the statistical analysis.

### Tongue-brushing – the X technique

**Figure 1:**
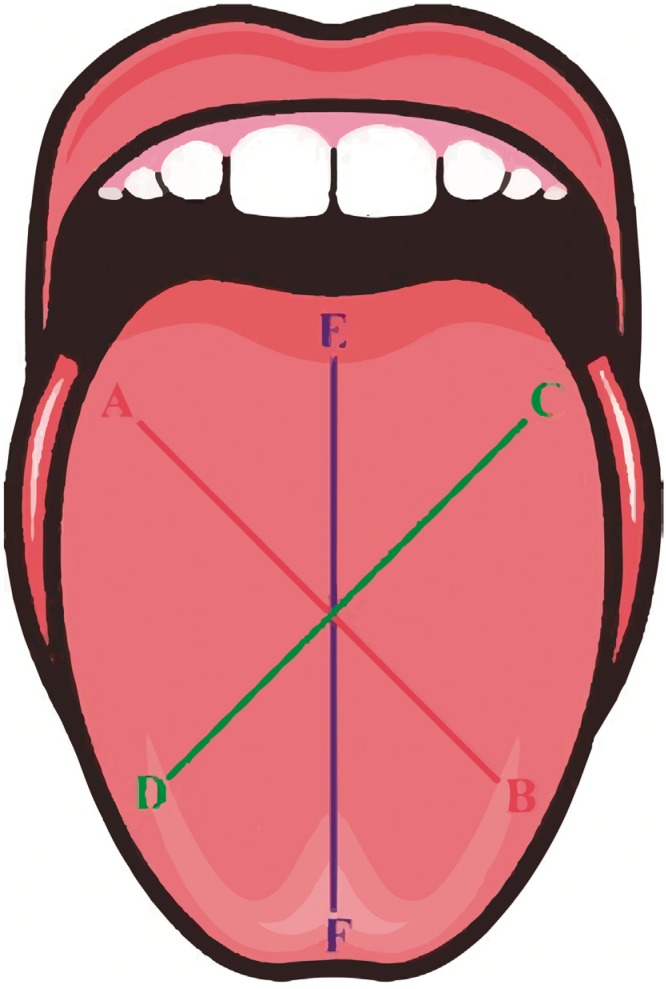
Movements involved in the execution of the X technique. First movement (A-B); second movement (C-D); third movement (E-F)

A new tongue-brushing technique was developed to systematically brush a large amount of the surface area of the tongue, which retains considerable quantities of substrates (shed epithelial cells, dead leucocytes). These substrates aid in the growth of microorganisms, so the technique can reduce the development of tongue coating and halitosis. The volunteers received a new toothbrush (Curaprox^®^ 5460 UltraSoft, Curaden, Switzerland) and were given systematic instructions on how to apply the tongue-brushing technique. The X technique involves three basic movements (Figure 1): after opening the mouth and extending the tongue, the patient positions the toothbrush (without toothpaste) on the posterior third of the tongue (in front of the vallate papillae) starting from the right side (Figure 1A). The patient then slides the bristles of the brush to the anterior region of the tongue in a transverse direction (Figure 1B). Next, the patient repeats the movement on the left side (Figure 1C and D). Finally, the patient positions the brush on the central region of the posterior third of the tongue (Figure 1E) and slides the bristles longitudinally toward the anterior edge (Figure 1F). After finishing the technique the brush was washed with water in abundance.

### Data analysis

The results are presented in tables and figures and consider absolute frequency, mean, median, minimum and maximum values, outliers, and quartiles. The paired numerical data were compared using Student's t-test for paired data. The ordinal paired data were compared using the Wilcoxon Signed Rank test. In the comparison of the three groups, the Kruskal-Wallis test and ANOVA were used. To correlate the scores on the VAS with the OAM results, Pearson's correlation test was applied. A 5% significance level was adopted in all of the tests. All of the numerical values exhibit normal distribution as *per* the Shapiro-Wilk test. All tests were performed by GraphPad Prism software (GraphPad Software, La Jolla California USA, version 7.00 for Windows,).

## Results and discussion

### Analysis of the organoleptic

**Figure 2:**
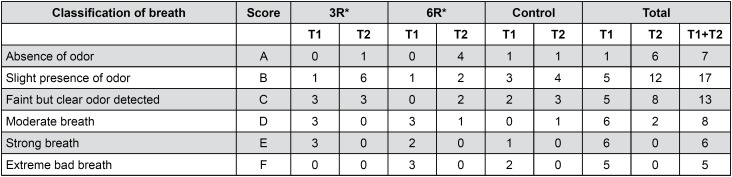
Organoleptic assessment scores for the three groups at the two consultations (T1: initial consolation; T2: two-week follow-up consultation). Results of the subjects’ organoleptic exams at the two consultations: T1 (initial examination) and T2 (two-week follow-up examination) in the three experimental groups: the 3R group, the 6R group, and the control group. The Wilcoxon signed-rank test revealed a statistically significant difference between T1 and T2 in the 3R group (p=0.011) and in the 6R group (p=0.007). The control group exhibited no differences between the two exams (p=0.071). The Kruskal-Wallis test revealed no statistically significant differences between the two test groups (p=0.531), a finding which was confirmed by ANOVA (p=0.577). The Wilcoxon single-ranked test. *Statistically significant difference; p<0.05

Assessment studies have shown that the mechanical methods commonly used to remove the coating on the tongue have a positive impact on reducing halitosis.^18^ ^,^ ^26^ ^,^ ^28^ In the comparison of each group's assessments from the initial consultation to those from the two-week follow-up consultation, a decrease was found in organoleptic scores in all of the groups. The lower scores indicate an improvement in most patients’ oral odor, despite the fact that they were allocated to different experimental groups. However, it is important to note that, regardless of the number of repetitions used in the X technique (three versus six), the application of the technique was associated with lower organoleptic scores relative to the control. In addition, patients in the 6R group achieved even lower organoleptic scores (Figure 2). The organoleptic assessment method is considered the gold standard in that it is cost free, simple, and practical; however, the examiner must be trained for accurate and consistent results to be obtained. In this study, the examiner was trained, and the scores produced by the examiner's evaluations were submitted to the Kappa test (0.7), the results of which justified the reliability of the diagnoses.

### Analysis of the Visual Analog Scale (VAS)

The question about individuals’ ability to detect their own halitosis using various techniques such as sniffing dental floss or saliva, licking the wrist and smelling it, or smelling the breath by placing the hand in front of the mouth and exhaling, led to many studies on the ability of self-perception of breath.^29^ ^,^ ^30^ Using a VAS, patients’ self-perception of their breath is compared to organoleptic assessments, levels of VSCs, laboratory tests, dental indices, and psychological profiles. The results suggest that people are, for the most part, unable to classify their own oral odor objectively.^31^ ^-^ ^33^ Patients who complain of halitosis do not always present this pathology, most patients suffering from halitophobia, and thus exhibited higher levels of dissatisfaction at the end of treatment. Psychological factors could explain the differences in the correlation between self-assessment and objective measures of halitosis;^33^ ^,^ ^34^ every patient has an idea of their own breath that varies according to their psychological profile. In the results of the VASs of this study, the patients in the 3R group and the 6R group reported an improvement in breath quality at the end of the study. According to the statistical analysis, the values found after three applications of the X technique demonstrated a significant reduction in perceptions of halitosis after the two-week study period (Table 1). This finding reflects the effectiveness of the technique in improving halitosis and the consequent benefits for patients’ social interactions and quality of life. Previous studies have suggested that the presence of the healthcare professional during the application of the VAS may influence the results, since the patient may feel intimidated by the professional to report improvements even if they are not true; patients may also be motivated to exhibit better commitment to their oral hygiene, knowing that it will be evaluated later. However, the patients in the control group, who, theoretically, would also suffer from the same bias, presented significantly worse values in the follow-up visit than in the initial consultation. These results, therefore, reinforce the effectiveness of the X technique.

**Table 1:** Visual analog scale (VAS) test presented by the mean of each of the three groups (3R, 6R, and control) at both consultations (T1: initial examination and T2: two-week follow-up examination)

Group	T1	T2	T1-T2	T2/T1	P value
3R	3.28	2.06	1.22	0.63	0.006*
6R	3.64	2.38	1.27	0.65	0.117
Control	3.3	4.01	-0.71	1.22	0.179

In absolute values, the control group exhibited an increase in the VAS test, as shown by the negative difference between T1 and T2. This finding shows that the patients in the control group did not experience self-perceived improvements in halitosis. The 3R group and the 6R group exhibited similar results in terms of their T2:T1 ratios (0.63 and 0.65, respectively). Student's paired t-test.

* Statistically significant difference; p<0.05

### Analysis of the microbiological

**Figure 3:**
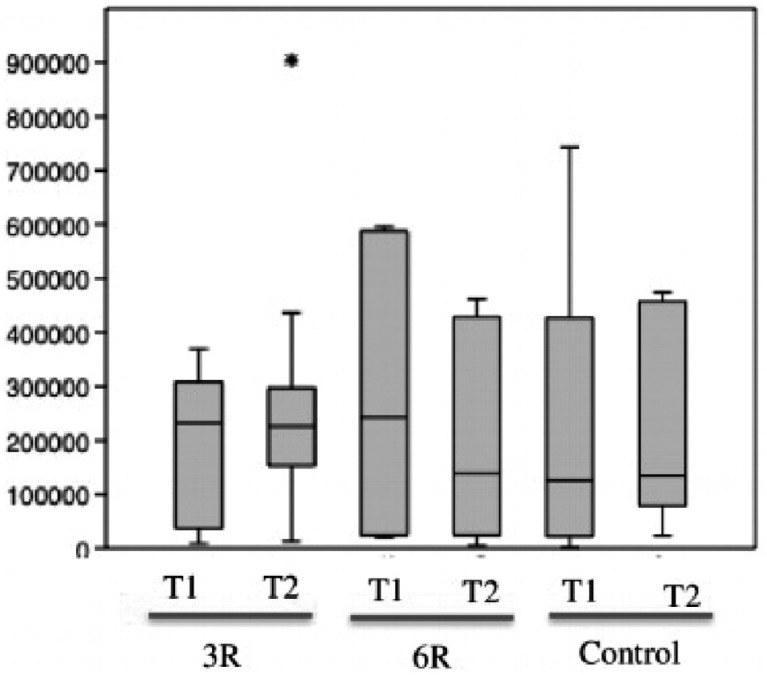
Boxplot of the number of colonies of the three groups at the two consultations (T1: initial examination and T2: two-week follow-up examination). Boxplot (median, minimum and maximum values, outliers, and quartiles). When the three groups’ T2 results were compared, the Kruskal-Wallis test and ANOVA revealed no significant differences between the numbers of colonies (p=0.577 and p=0.593, respectively). When the initial consultations (T1) and final consultations (T2) were compared, the 6R group exhibited a greater reduction in the number of bacterial colonies after the use of the X technique. In contrast, the 3R group exhibited a disparity: a greater number of bacterial colonies were found in T2 than in T1

In the samples of the microbiological analysis, no significant differences were found between the number of colonies present at the initial exam and the number of colonies present in the final two-week follow-up exam in any of the three groups. Although the other tests demonstrated a significant decrease in halitosis following the use of the technique, this decrease was not directly associated with the number of colonies present in the coating of the tongue. Studies have suggested that the amount of plaque on the tongue is directly correlated with halitosis.^35^ ^,^ ^36^ Authors such as Kazor et al.^37^ (2003) defined as an organoleptic score of 2 or more and volatile sulfur compound levels greater than 200 ppb. 16S rRNA genes from DNA isolated from tongue dorsum scrapings were amplified by PCR with universally conserved bacterial primers and cloned into Escherichia coli. Typically, 50 to 100 clones were analyzed from each subject. Fifty-one strains isolated from the tongue dorsa of healthy subjects were also analyzed. Partial sequences of approximately 500 bases of cloned inserts from the 16S rRNA genes of isolates were compared with sequences of known species or phylotypes to determine species identity or closest relatives. Nearly complete sequences of about 1,500 bases were obtained for potentially novel species or phylotypes. In an analysis of approximately 750 clones, 92 different bacterial species were identified. About half of the clones were identified as phylotypes, of which 29 were novel to the tongue microbiota. Fifty-one of the 92 species or phylotypes were detected in more than one subject. Those species most associated with healthy subjects were *Streptococcus salivarius, Rothia mucilaginosa*, and an uncharacterized species of *Eubacterium* (strain FTB41), however, state that the types of microorganisms present in the coating of the tongue may more clearly reflect the production of sulfur compounds and the consequent bad breath than simply the number of microorganisms. As with other oral pathologies, the authors indicate that halitosis may involve specific microbiological groups that exacerbate the problem. These bacteria typically include obligate Gram-negative and anaerobic species, such as *Peptostreptococcus anaerobius, Collinsella aerofaciens, Eubacterium group, Actinomyces spp., Eikenella corrodens, Veillonella spp., Fusobacteriumnucleatum*, pigmented *Prevotella spp.* and *Selenomonas spp.*, and there have been recent first-time reports of *Actinomyces turicensis, Collinsella aerofaciens, Eubacterium saburreum, E. timidum, Prevotella tannerae, Campylobacter concisus, Campylobacter mucosalis, Leptotrichia buccalis*, *Selenomonas flueggei*, and *Centipeda periodontii*.^35^ ^,^ ^38^ ^,^ ^39^ These bacteria belong to highly putrefactive groups and they are, therefore, able to cause the characteristic odor. In our analyses, we quantitatively evaluated the microorganisms present in the tongue coating. Future prospective studies should attempt to qualitatively investigate the coating on the tongue to determine which microorganisms may be responsible for worsening organoleptic scores, even if there are fewer colonies present. When each group's results from the initial consultations were compared to those from the respective final consultations, only the group that applied the technique six times (the 6R group) achieved a significant reduction in the number of bacterial colonies. In the control group, the number of microorganisms present in the final exam was significantly higher than the number found in the initial exam. These findings suggest that the lack of a standardized tongue brushing protocol results in the ineffective removal of the coating on the tongue. This superficial eviction of the organic substrate could be sufficient for reducing organoleptic scores, but it may not be effective in reducing the microorganisms that make up the coating (Figure 3). Though the results obtained in the organoleptic assessment were similar between the groups, the 3R group exhibited disparities in the number of bacterial colonies counted. More microorganisms were measured at the two-week follow-up consultation than at the initial consultation. This discrepancy is believed to be caused by the interference of the use of toothpaste when applying the technique. All of the patients were instructed on the importance of properly cleaning the toothbrush after brushing their teeth but prior to the execution of the technique. They were also advised not to use toothpaste when applying the technique to avoid both nausea and the accumulation of toothpaste on the dorsum of the tongue. In the samples of patients from the 3R group, we identified the presence of toothpaste in the samples, as well as subsequent fungal contamination, both of which were likely reflected in the microbiological analysis. Many people seek treatment for halitosis based on self-perception. Patients attempt to diagnose their own cases through various techniques or based on the perceptions of others in their social circles; their perceptions of halitosis are often influenced by psychological factors,^33^ which should therefore be considered. Halitosis is associated with several social problems: it can reduce an individual's quality of life as a result of embarrassment, communication difficulties and, in extreme cases, social isolation.^40^ The results obtained in this study are useful and thought-provoking. They suggest the need for additional complementary studies, which may include factors such as analyses of VSCs and salivary flow, qualitative microbiological analyses, or questionnaires to establish patients’ psychological profiles, as well as their hormone profiles, histories of periodontal disease, and other characteristics.

## Conclusions

The results of this study show that the systematic mechanical cleaning of the tongue using the X technique, especially when applied six times, has a significant positive effect on organoleptic scores and on the number of bacterial colonies present on the dorsum of the tongue. The technique also provides the sensation of improvement in breath according to patients’ self-perceptions.

## References

[1] Özen ME, Aydin M (2015). Subjective halitosis: definition and classification. J N J Dent Assoc.

[2] Kappor U, Sharma G, Juneja M, Nagpal A (2016). Halitosis: current concepts on etiology, diagnosis and management. Eur J Dent.

[3] Aydin M, Harvey-Woodworth CN (2016). Oral health: risk definition in halitosis. Br Dent J.

[4] Gaddey HL (2017). Oral manifestations of systemic disease. Gen Dent.

[5] Schumacher MG, Zürcher A, Filippi A (2017). Evaluation of a halitosis clinic over a period of eleven years. Swiss Dental J.

[6] Herman S, Lisowska G, Herman J, Wojtyna E, Misiolek M (2018). Genuine halitosis in patients with dental and laryngological etiologies of mouth odor: severity and role of oral hygiene behaviors. Eur J Oral Sci.

[7] Seerangaiyan K, van Winkelhoff AJ, Harmsen HJ, Rossen JW, Winkel EG (2017). The tongue microbiome in healthy subjects and patients with intra-oral halitosis. J Breath Res.

[8] Schemel-Suárez M (2017). Halitosis assessment and changes in volatile sulfur compounds after chewing gum: a study performed on Dentistry students. J Evid Based Dent Pract.

[9] Gondhalekar R, Richard KM, Jayachandra MG, Aslam S, Reddy VN, Barabde AS (2013). Effect of tongue cleaning methods and oral mutans streptococci level. J Contemp Dent Pract.

[10] Slot DE, De Geest S, van der Weijden FA, Quirynen M (2015). Treatment of oral malodour. Medium-term efficacy of mechanical and/or chemical agents: a systematic review. J Clin Periodontol.

[11] Sári KD, Kóródi A, Mohácsi R, Angyal J (2015). Oral health-related quality of life associations to periodontal conditions. Fogorv Sz.

[12] Tonzetich J (1977). Production and origin of oral malodor: a review of mechanisms and methods of analysis. J Periodontol.

[13] Seemann R, Duarte da Conceição M, Filippi A, Greenman J, Lenton P, Nachnani S (2014). Halitosis management by the general dental practitioner- results of an International Consensus Workshop. Swiss Dent J.

[14] Bernardi S, Marzo G, Continenza MA (2016). Dorsal lingual surface and halitosis: a morphological point of view. Acta Stomatol Croat.

[15] Uemori N, Kakinoki Y, Karaki J, Kakigawa H (2012). New method for determining surface roughness of tongue. Gerodontology.

[16] Roldán S, Herrera D, Sanz M (2003). Biofilms and the tongue: therapeutical approaches for the control of halitosis. Clin Oral Investig.

[17] Jamali Z, Aminabadi NA, Samiei M, Sighari Deljavan A, Shokravi M, Shirazi S (2016). Impact of chlorhexidine pretreatment followed by probiotic *Streptococcus salivarius* strain K12 on halitosis in children: a randomised controlled clinical trial. Oral Health Prev Dent.

[18] Van der Sluijs E, Slot DE, van der Weijden GA (2018). A PhD completed. Prevention and treatment of periodontal diseases and bad breath. Ned Tijdschr Tandheelkd.

[19] Feres M, Figueiredo LC, Faveri M, Guerra MC, Mateo LR, Stewart B (2015). The efficacy of two oral hygiene regimens in reducing oral malodour: a randomised clinical trial. Int Dent J.

[20] Asokan S, Kumar RS, Emmadi P, Raghuraman R, Sivakumar N (2011). Effect of oil pulling on halitosis and microorganisms causing halitosis: a randomized controlled pilot trial. J Indian Soc Pedod Prev Dent.

[21] Dudzik A, Chomyszyn-Gajewska M, Łazarz-Bartyzel K (2015). An evaluation of halitosis using Oral Chroma^TM^ Data Manager, organoleptic scores and patients’ subjective opinions. J Ind Soc Pedod Prev Dent.

[22] Greenman J, Lenton P, Seemann R, Nachnani S (2014). Organoleptic assessment of halitosis for dental professionals – general recommendations. J Breath Res.

[23] Bolepalli AC, Munireddy C, Peruka S, Polepalle T, Choudary Alluri LS, Mishaeel S (2015). Determining the association between oral malodor and periodontal disease: a case control study. J Int Soc Prev Community Dent.

[24] Brunner F, Kurmann M, Filippi A (2010). The correlation of organoleptic and instrumental halitosis measurements. Schweiz Monatsschr Zahnmed.

[25] Rosenberg M (1996). Clinical assessment of bad breath: current concepts. J Am Dent Assoc.

[26] Nandlal B, Shahikumar P, Avinash BS, Sreenivasan PK, Subramanyam R (2016). Malodor reductions and improved oral hygiene by toothbrushing and mouthrinsing. Indian J Dent Res..

[27] Danser MM, van Winkelhoff AJ, de Graaff J, Loos BG, van der Velden U (1994). Short-term effect of full-mouth extraction on periodontal pathogens colonizing the oral mucous membranes. J Clin Periodontol.

[28] Al-Maliky S, Hennequin-Hoenderdos NL, Slot DE, van der Sluijs E, Keijser BJ, van der Weijden GA (2016). Oral hygiene behaviour of a group of healthy students. Ned Tijdschr Tandheelkd.

[29] Yaegaki K, Coil JM (2000). Examination, classification, and treatment of halitosis; clinical perspectives. J Can Dent Assoc.

[30] Romano F, Pigella E, Guzzi N, Aimetti M (2010). Patients self-assessment of oral malodour and its relationship with organoleptic scores and oral conditions. Int J Dent Hyg.

[31] Yaegaki K, Coil JM (2000). Genuine halitosis, pseudo-halitosis, and halitophobia: classification, diagnosis, and treatment. Compend Contin Educ Dent.

[32] Oyetola OE, Owotade FJ, Fatusi OA, Olatunji S (2016). Pattern of presentation and outcome of routine dental interventions in patients with halitosis. Niger Postgrad Med J.

[33] Wang J, He L (2018). Comparison of the psychological condition of chinese patients with or without halitosis complaints. Chin J Dent Res.

[34] Suzuki N, Yoneda M, Naito T, Iwamoto T, Hirofuji T (2008). Relationship between halitosis and psychologic status. Oral Surg Oral Med Oral Pathol Oral Radiol Endod.

[35] Lee CH, Kho HS, Chung SC, Lee SW, Kim YK (2003). The relationship between volatile sulfur compounds and major halitosis-inducing factors. J Periodontol.

[36] LeBel G, Haas B, Adam AA, Veilleux MP, Lagha AB, Grenier D (2017). Effect of cinnamon (*Cinnamomum verum*) bark essential oil on the halitosis-associated bacterium *Solobacterium moorei* and *in vitro* cytotoxicity. Arch Oral Biol.

[37] Kazor CE, Mitchell PM, Lee AM, Stokes LN, Loesche WJ, Dewhirst FE (2003). Diversity of bacterial populations on the tongue dorsa of patients with halitosis and healthy patients. J Clin Microbiol.

[38] Takeuchi H, Machigashira M, Takeuchi N, Nakamura T, Noguchi K (2017). The association of periodontopathic bacteria levels in saliva and tongue coating with oral malodor in periodontitis patients. Oral Health Prev Dent.

[39] Rai M, Spratt D, Gomez-Pereira PR, Patel J, Nair SP (2016). Light activated antimicrobial agents can inactivate oral malodour causing bacteria. J Breath Res.

[40] Oho T, Yoshida Y, Shimazaki Y, Yamashita Y, Koga T (2001). Psychological condition of patients complaining of halitosis. J Dent.

